# Pelvic organ prolapse burden on sexual health and body image: a cross-sectional study

**DOI:** 10.1093/sexmed/qfaf100

**Published:** 2025-12-13

**Authors:** Roxana Geoffrion, Melissa Tigert, Jens-Erik Walter, May Sanaee, Erin A Brennand, Ola Malabarey, Maryse Larouche, Momoe Hyakutake, Fariba Mohtashami, Katherine Rabicki, Lina Roa, Terry Lee, Joel Singer, Nicole Koenig, Lori A Brotto

**Affiliations:** Department of Obstetrics & Gynecology, University of British Columbia, Vancouver, V6Z 2K8, Canada; Department of Obstetrics & Gynecology, University of British Columbia, Vancouver, V6Z 2K8, Canada; Department of Obstetrics and Gynecology, McGill University Montreal, Quebec, H3T 1M5, Canada; Department of Obstetrics & Gynecology, University of Alberta, Edmonton, T5G 0B6, Canada; Departments of Obstetrics & Gynecology and Community Health Sciences, Cumming School of Medicine, University of Calgary, Calgary, T2N 1N4, Canada; Department of Obstetrics & Gynecology, McMaster University, Hamilton, L8S 4K1, Canada; Department of Obstetrics and Gynecology, McGill University Montreal, Quebec, H3T 1M5, Canada; Department of Obstetrics & Gynecology, University of Alberta, Edmonton, T5G 0B6, Canada; Department of Obstetrics & Gynecology, University of British Columbia, Vancouver, V6Z 2K8, Canada; Department of Obstetrics & Gynecology, University of British Columbia, Vancouver, V6Z 2K8, Canada; Department of Obstetrics & Gynecology, University of British Columbia, Vancouver, V6Z 2K8, Canada; Centre for Advancing Health Outcomes, University of British Columbia, Vancouver, V6Z 1Y6, Canada; Centre for Advancing Health Outcomes, University of British Columbia, Vancouver, V6Z 1Y6, Canada; Department of Obstetrics & Gynecology, University of British Columbia, Vancouver, V6Z 2K8, Canada; Department of Obstetrics & Gynecology, University of British Columbia, Vancouver, V6Z 2K8, Canada

**Keywords:** female sexual health, body image, pelvic organ prolapse, pelvic floor disorders

## Abstract

**Background:**

Pelvic organ prolapse (POP) is common and can affect sexual health and body image. Sexual concerns of non-sexually active (NSA) females with POP have been poorly described.

**Aim:**

To describe the burden of POP and explore associations between sexual function and body image in all patients seeking surgery.

**Methods:**

Secondary analysis of a baseline patient cohort, randomized controlled trial (RCT) of 2 POP reconstructive surgeries at 5 urogynecology tertiary centers. We present baseline sexual activity, function, and sex-related affect and body image. Patients scheduled for POP surgery completed validated condition-specific questionnaires of sexual function, body image, demographics, POP burden, and medical comorbidities. We used descriptive statistics and appropriate tests of significance.

**Outcomes:**

Differences between sexually active (SA) and NSA patients in POP-specific sexual function and body image; associations between body image and impaired sexual function.

**Results:**

Of 181 patients, 86 (47.5%) were SA, with a mean (SD) of age 62 (11.1). SA patients were younger, with fewer comorbidities. 30/86 (35%) had clinically significant sexual function impairment. Reasons for being NSA included pelvic symptoms (69.1%), lack of interest (53.8%), and lack of partner (40.5%). Frustration, sexual inferiority, and anger were similar between NSA and SA patients. NSA patients have 5 times the odds of avoiding sexual activity because of fear of pelvic symptoms; twice the odds of being more dissatisfied and twice the odds of feeling more inadequate in their sex life than SA patients. (*P* < .05, unadjusted and adjusted analyses). A total of 171 patients (80% partnered) had similar body image scores between SA and NSA patients. Partnered NSA patients were more likely than SA to avoid sexual intimacy because of POP, aOR 2.35 (95% CI, 1.03-5.33). Patients who had clinically significant POP-related impairment of sexual function had significantly worse body image (*P* < .001).

**Clinical Implications:**

Patients with clinically bothersome POP have significant sexual concerns. Body image was similarly affected regardless of sexual activity. Perceived partner avoidance of intimacy was a common barrier. This is an opportunity for tailored sexual health individual and couples counseling.

**Strengths and Limitations:**

Our findings apply to many surgical POP patients regardless of partner or sexual activity status. Limitations include missing data, no validated measure of sexual distress, and no evaluation of partner sexual function.

**Conclusion:**

The burden of POP-related sexual dysfunction and corresponding poor body image is substantial in both SA and NSA patients awaiting POP surgery. In the preoperative assessment of POP, sexual health and body image questions should be routinely included.

## Introduction

According to the World Health Organization, “sexual health is fundamental to the overall health and well-being of individuals, couples and families”.^1^ Pelvic organ prolapse (POP) is an extremely prevalent pelvic floor disorder; it is estimated that up to 50% of parous women exhibit some degree of prolapse, and approximately 11% to 19% will require surgical correction during their lifetime.^2^^,^^3^

Sexual health, in the context of pelvic floor disorders in females, is a commonly avoided topic during clinical care, and is largely understudied and underrepresented in the literature.^4^ While physical symptoms of pelvic floor disorders, such as vaginal bulging, pressure, urinary or fecal incontinence, are well described, psychosocial impacts of these symptoms on sexual health and body image have only recently been studied.

Body image is a complex, multidimensional construct that reflects a person's perceptions, attitudes, and feelings about physical appearance. In female patients with POP, a deviation from normal pelvic support can significantly alter self-perception and lead to decreased body confidence and self-esteem.^5^^,^^6^ POP-related body image changes can contribute to broader psychological distress, including feelings of embarrassment, shame, and loss of femininity.^7^

While existing studies have primarily focused on sexually active (SA) female patients with pelvic floor disorders,^4^^,^^5^^,^^7^ a significant gap in the literature remains regarding those who engage in solo sexual activity or who are non-SA (NSA) due to choice, partner status, physical limitations, psychological or other concerns. These patients’ concerns are unclear, as they are often excluded from analyses of sexual function and its correlates. In order to design patient-centered clinical and sexual health counseling approaches, we need to first examine the complex interplay between specific POP-related symptoms of bulge or pressure and how they affect sexuality and body image in all POP patients regardless of partner or sexual activity status.

Our prespecified research question was: in female patients with symptomatic POP, seeking surgical correction, are there negative and sexual avoidance effects on sexual health and body image directly related to their pelvic floor disorder? Our objectives were to describe the burden of POP on facets of sexual health such as sexual activity, sexual function, and sex-related affect and body image, using validated tools. We assessed differences between SA patients compared to those who are NSA, and for partnered vs non-partnered SA patients. By exploring how these variables are related, we hope to advance a more inclusive, nuanced, and holistic framework for the clinical care of female patients affected by POP.

## Methods

We conducted a planned secondary analysis of baseline data from a double-blind, randomized, controlled, multicenter trial with a published detailed protocol.^8^ We registered the trial at clinicaltrials.gov (NCT02965313) and obtained institutional research ethics approval from all participating institutions (main site IRB# H16-02085). We conducted the trial from 2017 to 2024 in 5 Canadian tertiary care urogynecology centers and recruited female patients who failed or declined conservative POP management (pelvic floor muscle exercises or pessary) of uterine or vaginal vault POP. The main RCT trial design helped to eliminate some common sources of bias, and all baseline patients with available data were included in this cross-sectional study, with minimal sampling, recall, or attrition bias. Patients had availability of translation for questionnaire responses, helping to mitigate language bias. Data was systematically collected and entered into an electronic database as it became available. All patients had significantly bothersome symptoms of protrusion, bulge, or pressure warranting a reconstructive, minimally invasive vaginal surgery, including hysterectomy and POP repairs. All surgeries were performed through a vaginal approach, with standardized technique, including a posthysterectomy vaginal vault suspension to the sacrospinous ligaments with synthetic suture or synthetic mesh arms (patients randomized to either group). Exclusion criteria were: patients wishing to conserve the uterus; prior pelvic radiation; prior pelvic mesh or concurrent need for synthetic vaginal mesh other than incontinence sling; vaginal pain (muscle spasm/tenderness with palpation of muscle groups behind the vagina); patient-expressed inability to follow up; chronic pain conditions; smoking or active immunosuppressant use or other reason for immune suppression in the 3 months prior to surgery. Sample size was based on a predefined composite primary outcome 2 years after surgery.^8^ At baseline, we collected demographic data and detailed pelvic examinations with objective staging of POP severity on a scale of 0 (no POP) to 4 (severe POP).^9^

We investigated sexual function through a questionnaire validated in patients with POP (POP/Incontinence Sexual Questionnaire, IUGA-Revised—PISQ-IR).^10^^,^^11^ PISQ-IR explores the physical and emotional impact of pelvic floor disorders, personal health, partner impact, and personal sexual interest impact on sexual health through subscales that are different in SA and NSA patients. It also explores sexual quality, satisfaction (for both SA and NSA), and desire, arousal, orgasm, pain (for SA only). It allows analysis of SA patients without a partner and incorporates gender-neutral questions.^12^ Additionally, for SA patients, it has a summary score cutoff value calculated at 2.68, with values under 2.68 indicating impaired sexual function.^12^ We defined “SA” based on individual response to question one of the PISQ-IR: either “NSA at all” or “SA with or without a partner.”

We investigated body image through a questionnaire validated in patients with POP (Body Image in POP Questionnaire—BIPOP).^13^ BIPOP has total, attractiveness and partner subscales, with Likert scale answers of “strongly agree” (scored as 5) to “strongly disagree” (scored as 1) and higher scores indicating worse body image.^13^ It has different versions for partnered and non-partnered women with slightly different phrasing. We differentiated between “partnered” or “non-partnered” based on which version the patient selected to answer.

In addition, we assessed patient comorbid health conditions via the Charlson Comorbidity Index^14^; health-related quality of life via the EQ-5D^15^^,^^16^; and severity of pelvic floor disorder impact via validated condition-specific quality of life questionnaires (Pelvic Floor Distress Inventory—PFDI-20 and Pelvic Floor Impact Questionnaire—PFIQ-7^17^).

We used descriptive statistics to summarize these data and compared them between SA and NSA and between partnered and non-partnered patients using *t*-tests, Wilcoxon rank sum tests, chi-square tests, or Fisher’s exact test as appropriate. We further used ordinal regression to compare PISQ-IR and BIPOP questions between these groups while adjusting for baseline characteristics which were different between the groups (*P* < .05). Proportional odds assumption was checked and was satisfied in all analyses. Variance inflation factor was examined and no multicollinearity was detected in the regression analysis. Patients with missing data were excluded from the corresponding analysis. All data were analyzed with Statistical Analysis System (SAS) software version 9.4 (SAS Institute).

## Results

**Figure 1:**
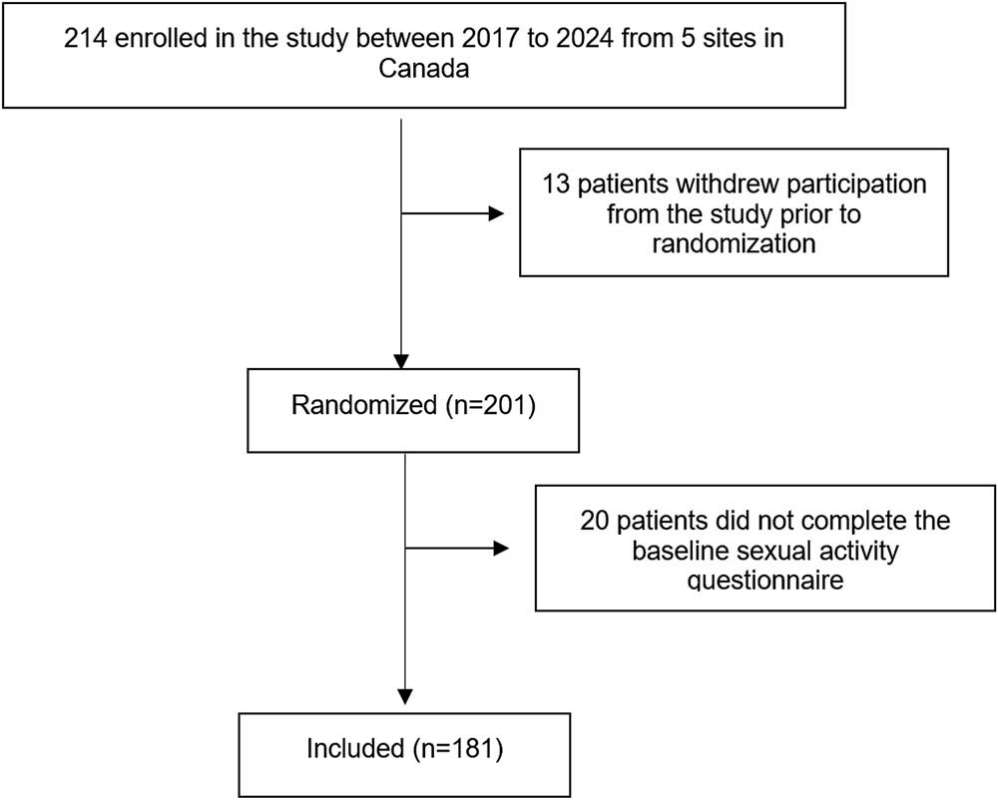
Study flow chart.

A total of 201 patients were randomized in the main RCT and baseline sexual activity data were available for 181 patients (90%; Figure 1). Our cohort had a mean age of 62 and a mean BMI of 28, and most patients were postmenopausal (76%). Most patients had stage 2 or higher POP (99.4%), with significant bother on condition-specific pelvic floor disorders questionnaires (Table 1).

**Table 1:** Baseline demographics in all, NSA and SA patients.

	**All (*n* = 181)**	**NSA (*n* = 95)**	**SA (*n* = 86)**	***P***
Age, mean (SD)	62.3 (11.1)	66.4 (8.5)	57.9 (11.9)	<.001
BMI, mean (SD)	28.0 (5.3)	28.5 (5.3)	27.7 (5.3)	.354
Missing, *n*	30	22	8	
Menopausal, *n* (%)	135/178 (75.8)	81/94 (86.2)	54/84 (64.3)	.001
Vaginal estrogen, *n* (%)	44/172 (25.6)	23/88 (26.1)	21/84 (25.0)	.864
Chronic constipation >6 months, *n* (%)	18/175 (10.3)	10/90 (11.1)	8/85 (9.4)	.711
Low back pain >6 months, *n* (%)	22/174 (12.6)	16/91 (17.6)	6/83 (7.2)	.040
Maximum Prolapse Stage—apical, anterior, and posterior, mean (SD)	2.9 (0.9)	2.9 (0.8)	2.8 (0.9)	.394
Comorbidities, *n* (%)				
Asthma, emphysema, or chronic bronchitis	12/179 (6.7)	9/93 (9.7)	3/86 (3.5)	.098
Arthritis or rheumatism	55 (30.4)	37 (38.9)	18 (20.9)	.008
Cancer, diagnosed in the past 3 years	7/180 (3.9)	4/94 (4.3)	3/86 (3.5)	1.000
Diabetes	21 (11.6)	11 (11.6)	10 (11.6)	.992
Digestive problems	9/180 (5.0)	5/94 (5.3)	4/86 (4.7)	1.000
Heart trouble	5 (2.8)	4 (4.2)	1 (1.2)	.371
Kidney disease	2/180 (1.1)	1/94 (1.1)	1/86 (1.2)	1.000
Liver problems	2/178 (1.1)	1/92 (1.1)	1/86 (1.2)	1.000
Stroke	1/177 (0.6)	1/91 (1.1)	0/86 (0.0)	1.000
Number of above comorbidities, *n* (%)				<.001
Unknown	7	7	0	
0	96 (55.2)	37 (42.0)	59 (68.6)	
1	52 (29.9)	36 (40.9)	16 (18.6)	
2	24 (13.8)	15 (17.0)	9 (10.5)	
3	1 (0.6)	0 (0.0)	1 (1.2)	
4	1 (0.6)	0 (0.0)	1 (1.2)	
PFDI, median (IQR)	124.0 (75.0, 165.6)	129.2 (76.0, 169.8)	115.1 (72.9, 159.4)	.331
PFIQ, median (IQR)	64.3 (23.8, 133.3)	56.3 (19.0, 142.9)	69.0 (28.6, 114.3)	.838
EQ-5D-3L—Some/extreme problems, *n* (%)				
Mobility	52/172 (30.2)	35/92 (38.0)	17/80 (21.3)	.017
Self-care	10/174 (5.7)	8/92 (8.7)	2/82 (2.4)	.077
Usual activities	60/170 (35.3)	34/90 (37.8)	26/80 (32.5)	.472
Pain/discomfort	126/173 (72.8)	68/91 (74.7)	58/82 (70.7)	.555
Anxiety/depression	80/175 (45.7)	46/92 (50.0)	34/83 (41.0)	.231
EQ-5D-3L—VAS, mean (SD)	71.8 (16.7)	72.2 (17.9)	71.3 (15.5)	.731
Missing, *n*	10	4	6	

NSA, non-sexually active; SA, sexually active; PFDI, pelvic floor distress inventory; PFIQ, pelvic floor impact questionnaire.

### Sexual function, SA patients

**Figure 2:**
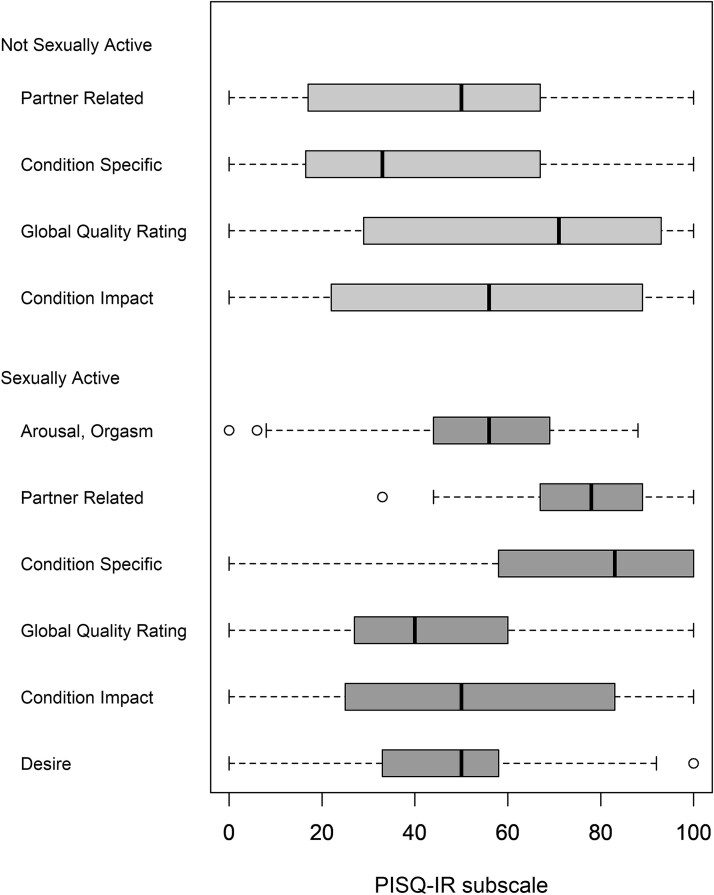
PISQ-IR subscale scores in NSA and SA patients. NSA, non-sexually active; SA, sexually active.

86/181 patients (47.5%) were SA at baseline, among whom only 5 had no sexual partner, which precluded analysis of the solo sexual activity group separately. SA patients were younger (*P* < .001), with fewer comorbidities (*P* < .001), when compared to NSA patients. They had a median (IQR, range) PISQ-IR score of 3.0 (2.5-3.5, 1.5-4.2) (subscale scores in Figure 2 and missing data in Table S1). 29/82 (35%) had a score of less than 2.68, indicating clinically significant sexual concerns. Almost half of SA patients (47%) had daily or weekly sexual thoughts, and 46.3% reported moderate sexual desire or interest. Dyspareunia was present “usually” or “always” in 17.1% of SA patients.

### Sexual function, NSA patients

95/181 were NSA because of (response of “strongly” or “somewhat agree”): pelvic floor symptoms (*N* = 56/81, 69.1%), lack of interest in sex (*N* = 42/78, 53.8%), lack of partner (*N* = 32/79, 40.5%), pain (*N* = 27/72, 37.5%), or other health problems (*N* = 14/75, 18.7%). Some of the 95 patients chose not to respond to some of the individual questions, resulting in different denominators. 60/88 NSA patients (68.2%) expressed avoiding or restricting sexual activity specifically because of fear of pelvic symptoms. 50/78 patients (64.1%) expressed dissatisfaction with their sex lives and 49/76 (64.5%) expressed inadequacy with their sex lives. NSA patients felt frustrated (*N* = 51/87, 58.6%), sexually inferior (*N* = 48/86, 55.8%), and angry (*N* = 40/85, 47.1%) about sexual inactivity related to pelvic floor disorders. About 25.8% experienced “a lot” of bother because of sexual inactivity.

### Comparison of sexual function between SA and NSA active patients

**Figure 3:**
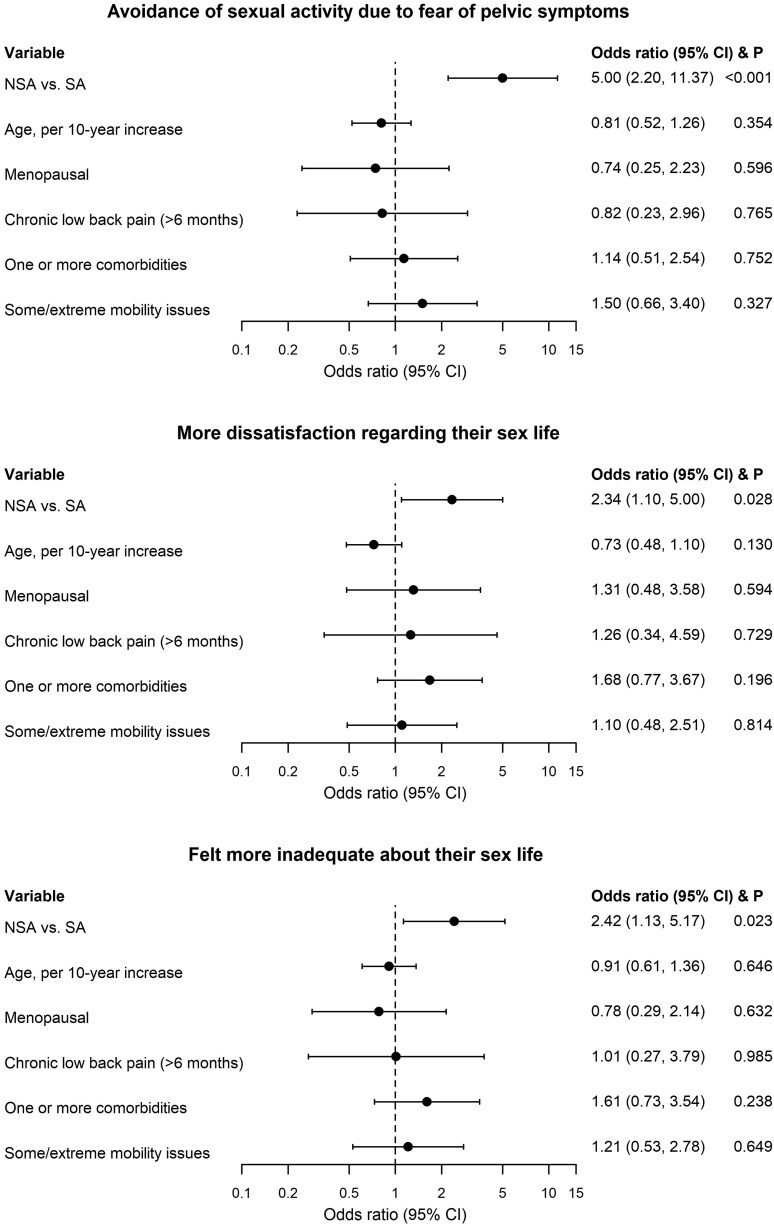
Comparison of sexual function by multivariable ordinal regression.

Frustration, sexual inferiority, and anger were similar between NSA and SA patients. NSA patients were “a lot” more avoidant of sexual activity because of fear of pelvic symptoms (OR (95% CI): 3.18 (1.67-6.04), *P* < .001); reported more dissatisfaction regarding their sex life (OR (95% CI): 2.14 (1.16-3.96), *P* = .016); and felt more inadequate about their sex life (OR (95% CI): 2.43 (1.30-4.54), *P* = .005) than SA patients. These findings remained the same after adjusting for age, menopausal status, number of comorbidities, presence of chronic low back pain (>6 months), and mobility issues (aOR (95% CI): avoidance of sexual activity due to fear of pelvic symptoms: 5.00 (2.20-11.37), *P* < .001; dissatisfaction: 2.34 (1.10-5.00), *P* = .028; inadequacy feeling: 2.42 (1.13-5.17), *P* = .023; Figure 3). Within the subgroup of menopausal women, similar results were observed (aOR (95% CI): avoidance: 4.26 (1.74-10.42), *P* = .002; dissatisfaction: 2.12 (0.91-4.93), *P* = .083; inadequacy feeling: 2.29 (0.98-5.38), *P* = .056). When stratified the analysis by age (<65 vs ≥65), the adjusted effect size (ie, aOR) in these 2 subgroups was comparable to the analysis using the full cohort.

### Body image

171/181 patients (94.5%) completed the BIPOP questionnaire, among whom 137/171 (80.1%) were partnered, including 80 SA and 57 NSA. There was a wide range of body image scores. There were no differences in total BIPOP, attractiveness, and partner subscales between SA and NSA in partnered or non-partnered patients (Table 2). When individual question responses were compared, there was a significant difference between SA and NSA partnered patients in worry about the partner avoiding intimacy, with NSA being more worried (OR (95% CI): 1.87 (1.01-3.43), *P* = .045). When adjusted for age, menopausal status, number of comorbidities, back pain, and mobility issues, the result changed marginally (aOR (95% CI): 2.16 (0.98-4.75), *P* = .055). There was also a significant difference in the patient avoidance of intimacy because of POP, with NSA being more avoidant (OR (95% CI): 2.25 (1.14-4.47), *P* = .020). When adjusted for the same aforementioned potential confounders, this retained significance (aOR (95% CI): 2.35 (1.03-5.33), *P* = .041).

**Table 2:** Body image scores in all, NSA and SA patients, both partnered and non-partnered.

**Partnered** ^a^	**All (*n* = 137)**	**NSA (*n* = 57)**	**SA (*n* = 80)**	**Difference (95% CI)**	***P***
BIPOP Attractiveness Subscale				0.2 (−0.2 to 0.6)	.265
Mean (SD)	3.5 (1.2)	3.6 (1.2)	3.4 (1.2)		
Range	(1.0, 5.0)	(1.0, 5.0)	(1.0, 5.0)		
BIPOP Partner Subscale				0.3 (−0.1 to 0.7)	.125
Mean (SD)	3.4 (1.2)	3.6 (1.1)	3.3 (1.2)		
Range	(1.0, 5.0)	(1.0, 5.0)	(1.0, 5.0)		
BIPOP total				0.3 (−0.1 to 0.7)	.158
Mean (SD)	3.5 (1.1)	3.6 (1.1)	3.3 (1.1)		
Range	(1.0, 5.0)	(1.0, 5.0)	(1.0, 5.0)		
**Non-partnered** ^a^	**All (*n* = 34)**	**NSA (*n* = 29)**	**SA (*n* = 5)**		***P***
BIPOP Attractiveness Subscale				0.1 (−1.5 to 1.2)	.821
Mean (SD)	3.4 (1.3)	3.4 (1.4)	3.5 (1.1)		
Range	(1.0, 5.0)	(1.0, 5.0)	(1.8, 4.6)		
BIPOP Partner Subscale				−0.4 (−1.6 to 0.8)	.502
Mean (SD)	3.7 (1.2)	3.7 (1.3)	4.1 (0.7)		
Range	(1.0, 5.0)	(1.0, 5.0)	(3.2, 4.8)		
BIPOP total				0.3 (−1.5 to 0.9)	.646
Mean (SD)	3.6 (1.2)	3.5 (1.3)	3.8 (0.9)		
Range	(1.0, 5.0)	(1.0, 5.0)	(2.5, 4.7)		

^a^Ten patients (5.5%) did not complete the BIPOP questionnaire. For partnered women, BIPOP Attractiveness Subscale was missing for 1 SA patient.

We also examined individual responses to BIPOP in partnered vs non-partnered patients regardless of sexual activity, and there were no differences in total BIPOP, attractiveness, and partner subscales. When individual question responses were compared, there was a significant difference between partnered and non-partnered patients in worry about the partner avoiding intimacy, with P patients being more worried (OR (95% CI): 2.87 (1.31-6.28), *P* = .028). When adjusted for age (the only baseline demographics that was significantly different between partnered and non-partnered patients), this retained significance (aOR (95% CI): 3.59 (1.57-8.20), *P* = .018).

Among partnered NSA patients, body image was significantly worse in total score (mean (SD): 3.8 (1.0) vs 3.0 (1.3); *P* = .037) and partner subscale score (mean (SD): 3.8 (1.1) vs 2.9 (1.4); *P* = 0.017) in patients who “strongly agreed” and “somewhat agreed” that lack of sexual activity is directly related to pelvic floor symptoms when compared to patients who did not (PISQ-IR question 2c).

Partnered SA patients who had a PISQ-IR score in the range for clinically significant impairment of sexual function (<2.68) had significantly worse body image for attractiveness (mean (SD): 4.1 (0.8) vs 3.0 (1.2), *P* < .001) and partner subscales (mean (SD): 4.1 (0.7) vs 2.8 (1.2), *P* < .001), as well as for total BIPOP score (mean (SD): 4.1 (0.6) vs 2.9 (1.1), *P* < .001) than those without impairment. Also, there were moderate negative correlations between BIPOP scores and continuous scale PISQ-IR scores (Pearson correlation coefficient = −0.53, −0.65, −0.63 for attractiveness, partner, and total BIPOP scores).

## Discussion

Our study presents a cross-sectional picture of the burden of POP and related symptoms on female sexual health and body image in patients recruited across Canada into a randomized controlled trial and awaiting surgery for POP. Our trial had rigorous data collection, using sexual function questionnaires validated for patients with pelvic floor disorders, in multiple Canadian sites, and thus provided a good opportunity for our planned secondary analysis of the burden of baseline sexual dysfunction in patients seeking surgical POP repair. We showed significant sexual concerns in both SA and NSA patients. Body image was similarly affected regardless of sexual activity. A majority of NSA patients expressed avoiding or restricting sexual activity specifically because of fear of pelvic symptoms, with feelings of sexual avoidance, fear, dissatisfaction, and inadequacy emerging as significant. Perceived partner avoidance of intimacy was identified as a barrier. To our knowledge, this is the largest cross-sectional study to highlight both sexual dysfunction and body image concerns in SA and NSA patients with POP and concurrent related pelvic symptoms.

While sexual function is multifactorial, encompassing physical, psychological, and interpersonal elements, POP in particular may interfere with sexual activity through both anatomical disruption and emotional inhibition. Commonly reported concerns include decreased libido, pain with intercourse, reduced sexual satisfaction, and fear of worsening the POP during intimacy.^18^^,^^19^ Since the patients in our study were carefully selected to exclude other conditions causing chronic pelvic pain and muscle spasm (contraindication to vaginal surgery), few patients had dyspareunia. The proportion of our patients with pain (17.1%) likely represents the prevalence of POP-related sexual pain. Similar to another cross-sectional study of patients presenting with pelvic floor complaints,^20^ in our SA patients, the subdomains with the lowest PISQ-IR scores and thus the highest impact on sexual function were global quality (most affected) and desire and condition impact (second-most affected). Approximately half of both SA and NSA patients in our study had moderate sexual interest. This is perhaps associated with a cohort contemplating reconstructive surgery and hoping for an improvement in sexual function through POP resolution. POP and fecal symptoms of obstruction and incontinence were associated with not enjoying sex in another cross-sectional study.^21^ However, when adjusted for other determinants of sexual dysfunction (eg, aging, dyspareunia, vaginal atrophy, partner issues), these associations were no longer significant. The questionnaire used to assess sexual function in their study has been validated for SA participants only and did not address concerns of NSA patients.

Many NSA patients (57/86, 66.3%) had a partner in our cohort. Sexual dissatisfaction and sexual inadequacy were at least twice more likely in this group than in the SA patients. NSA patients, as well as partnered patients who filled out the body image assessment, were overall more worried about their partner being avoidant of intercourse. There are very few studies attempting to quantify the sexual experiences of partners of patients with pelvic floor disorders. Preoperative sexual function of male partners in a small cohort of patients undergoing surgery for POP and urinary incontinence was not statistically different compared to population estimates, adjusted for age.^22^ Postoperatively, male partners’ sexual function was unchanged or mildly improved. A mixed methods exploration of the circular sexual response cycle in women with POP identified themes similar to our findings, with participants reporting a drastic decrease in willingness to initiate sex or respond to sexual invitations since becoming aware of their POP, sometimes because of fear their partner would be “grossed out” by their POP.^23^ In this study, supportive partners did help attenuate distress and embarrassment, suggesting a possible therapeutic role for open communication and couples sexual therapy counseling or cognitive behavioral therapy.

Negative body image has been independently associated with sexual dysfunction in postmenopausal women in general.^24^ Our study presents particular perspectives of patients who are both postmenopausal (the majority of our cohort) and have significantly bothersome POP, while identifying the association between worse body image scores and clinically significant sexual dysfunction. In our study, we found that avoidance of sexual intimacy was a common theme from both sexual function (PISQ-IR scores) and body image (BIPOP score) assessments. Other qualitative studies highlight the impact of shame and stigma associated with pelvic floor disorders on health and recovery. This needs to be addressed in clinical counseling for sexual health of patients suffering from pelvic floor disorders. Body image assessment is a useful tool in the context of managing POP, particularly as it relates to sexual health.

### Strengths and limitations

One of the strengths of our study is its broad geographic distribution across Canada, with a large number of patients recruited in several centers. We used meticulous data collection at each institution and our validated questionnaires addressed various domains of sexual function and body image, inclusive of patients with or without sexual activity. Unlike other studies, our research empowered NSA patients to voice and quantify their intimacy-related concerns. Comprehensive demographic variable collection allowed us to conduct a meaningful regression analysis to correct for baseline confounders and present a cross-sectional overview of the impact of POP on sexuality.

The choice of main instrument to evaluate sexual function, the PISQ-IR, is both a strength and a limitation. While we wished to describe POP-related sexual concerns in detail, we also recognize this limits our ability to describe sexual dysfunction based on other recognized domains such as lack of lubrication or arousal. While the PISQ-IR does measure the emotional impact of sexual difficulties, we did not evaluate sexual distress with a separate validated scale. While the PISQ-IR does address negative emotions of poor sexual health, there is no sexual distress scale validated in this patient population.

Other limitations of our study include the lack of generalizability to other surgical populations (eg, pelvic pain patients or those wishing uterine preservation in the treatment of POP), missing data due to the sensitive nature of questions and assessment of sexual function in a generational cohort historically not inclined to share sexual experiences. We found that even within the same questionnaire, patients chose to respond to certain questions but not others, indicating a potential level of discomfort with individual questions and possibly introducing responder bias. We did not have sufficient diversity within our study to evaluate cultural, religious, or other identity barriers to sexual health in patients with pelvic floor disorders. We did not capture use of drugs or collect mental health information about clinical depression or anxiety, for example; these comorbidities could affect sexuality. We also did not evaluate partner sexual function. As sexual function is highly dependent on partner interaction, the lack of partner-related variables is an important limitation. However, several studies of partners of patients with pelvic floor disorders do not show a strong impact of these disorders on partner aspects of sexual function.^22^^,^^23^ Future research could introduce partner-matching questionnaires and qualitative research methods could also be used to form a more comprehensive understanding of couples’ sexual health in this context.

## Conclusion

In conclusion, our study suggests a significant burden of POP-related sexual dysfunction, which we showed to be substantial in both SA and NSA patients awaiting POP surgery, with notable differences in body image. Our findings supplement the world literature on aging female sexual health by including patients who were NSA and by exploring their reasons for sexual inactivity, as well as their emotional state associated with lack of physical intimacy. Our study therefore addresses a critical gap in understanding an often-overlooked patient population. This broader perspective better informs clinicians during initial consultations and helps them address sexual health concerns. In the preoperative assessment of POP, sexual health and body image questions should be routinely included. For NSA patients in particular, we recommend clinicians proactively inquire about reasons for sexual inactivity and related level of bother in order to provide referrals for sexual health counseling according to individual needs. Our findings are directly clinically relevant and applicable to individual treatment plans. Future research from our group will examine how surgical treatment for POP affects sexual health and body image, with potential improvements likely to influence baseline counseling regarding POP treatments.

## Supplementary Material

COMET_Body_Image_TABLE_2_supplement_revised_qfaf100

## Notes

**Conference Presentations:** Poster at the American Urogynecologic Society annual meeting, Vancouver, Canada, October 2025.
