# Supplementary material for: Pelvic organ prolapse burden on sexual health and body image: a cross-sectional study
Source: Sex Med. 2025 Dec 13;13(6):qfaf100. doi: 10.1093/sexmed/qfaf100 (PMC12780765; doi:10.1093/sexmed/qfaf100)
Supplement: COMET_Body_Image_TABLE_2_supplement_revised_qfaf100 [file comet_body_image_table_2_supplement_revised_qfaf100.docx]

Supplement Table: Sexual function scores in NSA and SA patients, including missing data

|  | **NSA (n=95)** | **SA (n=86)** |
| --- | --- | --- |
| PISQ |  |  |
| Missing, n | - | 4 |
| Median (IQR) | - | 3.0 (2.5, 3.5) |
| Range | - | (1.5, 4.2) |
| NSA-PR: Not Sexually Active - Partner Related |  |  |
| Missing, n | 12 | - |
| Median (IQR) | 50.0 (17.0, 67.0) | - |
| Range | (0.0, 100.0) | - |
| NSA-CS: Not sexually active - Condition Specific |  |  |
| Missing, n | 19 | - |
| Median (IQR) | 33.0 (16.5, 67.0) | - |
| Range | (0.0, 100.0) | - |
| NSA-GQA: Not sexually active - Global Quality Rating |  |  |
| Missing, n | 6 | - |
| Median (IQR) | 71.0 (29.0, 93.0) | - |
| Range | (0.0, 100.0) | - |
| NSA-CI: Not sexually active - Condition Impact |  |  |
| Missing, n | 9 | - |
| Median (IQR) | 56.0 (22.0, 89.0) | - |
| Range | (0.0, 100.0) | - |
| SA-AO: Sexually Active: Arousal, Orgasm |  |  |
| Missing, n | - | 3 |
| Median (IQR) | - | 56.0 (44.0, 69.0) |
| Range | - | (0.0, 88.0) |
| SA-PR: Sexually Active: Partner Related |  |  |
| Missing, n | - | 16 |
| Median (IQR) | - | 78.0 (67.0, 89.0) |
| Range | - | (33.0, 100.0) |
| SA-CS: Sexually Active: Condition Specific |  |  |
| Missing, n | - | 5 |
| Median (IQR) | - | 83.0 (58.0, 100.0) |
| Range | - | (0.0, 100.0) |
| SA-GQ: Sexually Active: Global Quality |  |  |
| Missing, n | - | 9 |
| Median (IQR) | - | 40.0 (27.0, 60.0) |
| Range | - | (0.0, 100.0) |
| SA-CI: Sexually Active: Condition Impact |  |  |
| Missing, n | - | 5 |
| Median (IQR) | - | 50.0 (25.0, 83.0) |
| Range | - | (0.0, 100.0) |
| SA-D: Sexually Active: Desire |  |  |
| Missing, n | - | 3 |
| Median (IQR) | - | 50.0 (33.0, 58.0) |
| Range | - | (0.0, 100.0) |
